# Natural Sulfur-Containing Compounds: An Alternative Therapeutic Strategy against Liver Fibrosis

**DOI:** 10.3390/cells8111356

**Published:** 2019-10-30

**Authors:** Alfonsina Milito, Mariarita Brancaccio, Giuseppe D’Argenio, Immacolata Castellano

**Affiliations:** 1 Department of Biology and Evolution of Marine Organisms, Stazione Zoologica Anton Dohrn, Villa Comunale, 80121 Naples, Italy; alfonsina.milito@szn.it (A.M.); mariarita.brancaccio@szn.it (M.B.); 2 Department of Clinical Medicine and Surgery, Gastroenterology Unit, School of Medicine, Federico II University, Via Pansini, 5, 80131 Naples, Italy; dargenio@unina.it

**Keywords:** liver fibrosis, natural products, sulfur-containing compounds, glutathione, sulforaphane, lipoic acid, taurine, ergothioneine, ovothiol, garlic

## Abstract

Liver fibrosis is a pathophysiologic process involving the accumulation of extracellular matrix proteins as collagen deposition. Advanced liver fibrosis can evolve in cirrhosis, portal hypertension and often requires liver transplantation. At the cellular level, hepatic fibrosis involves the activation of hepatic stellate cells and their transdifferentiation into myofibroblasts. Numerous pro-fibrogenic mediators including the transforming growth factor-β1, the platelet-derived growth factor, endothelin-1, toll-like receptor 4, and reactive oxygen species are key players in this process. Knowledge of the cellular and molecular mechanisms underlying hepatic fibrosis development need to be extended to find novel therapeutic strategies. Antifibrotic therapies aim to inhibit the accumulation of fibrogenic cells and/or prevent the deposition of extracellular matrix proteins. Natural products from terrestrial and marine sources, including sulfur-containing compounds, exhibit promising activities for the treatment of fibrotic pathology. Although many therapeutic interventions are effective in experimental models of liver fibrosis, their efficacy and safety in humans are largely unknown. This review aims to provide a reference collection on experimentally tested natural anti-fibrotic compounds, with particular attention on sulfur-containing molecules. Their chemical structure, sources, mode of action, molecular targets, and pharmacological activity in the treatment of liver disease will be discussed.

## 1. Introduction

**Figure 1:**
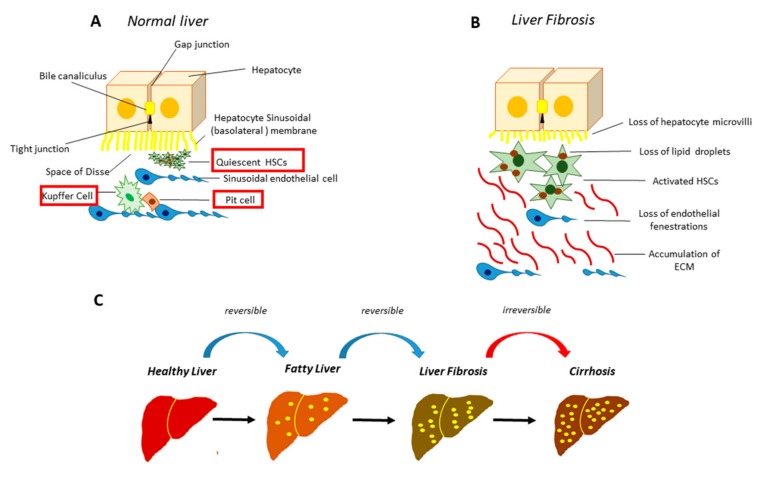
Normal and fibrotic liver architecture. (**A**) In the healthy liver the hepatocytes are closely joined by gap junctions. The liver sinusoid, a large fenestrated capillary, is mainly constituted by sinusoidal endothelial cells (SECs), Kupffer cells, specialized hepatic macrophages, and the Pit cells, the natural killer lymphocytes of the liver. The space between the hepatocytes and the sinusoid, called “the space of Disse”, hosts the hepatic stellate cells (HSCs); (**B**) following HSCs activation, there is a massive accumulation of extracellular matrix (ECM) as well as the loss of microvilli in the hepatocytes and the loss of fenestrae in SECs; (**C**) stages of hepatic fibrosis.

**Figure 2:**
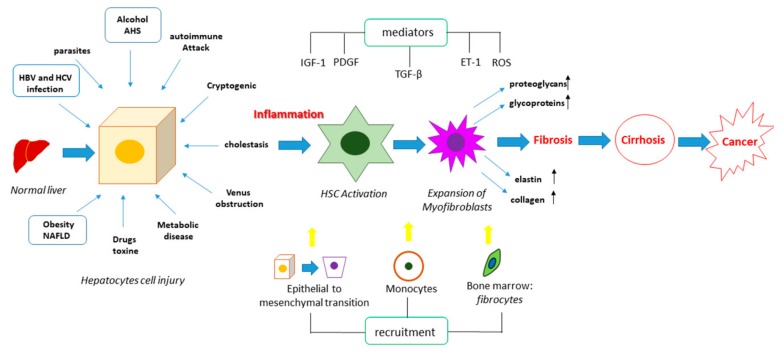
Pathogenesis of liver fibrosis. Liver fibrosis can be induced by different insults, which can trigger cell death processes in the hepatocytes, leading to the release of pro-fibrogenic mediators such as the transforming growth factor beta (TGF-β), the platelet-derived growth factor (PDGF), insulin-like growth factor-1 (IGF-1), endothelin 1 (ET-1), and reactive oxygen species (ROS). These can activate HSCs, promoting their differentiation into myofibroblasts, as well as they can stimulate the epithelial to mesenchymal transition and the recruitment of circulating monocytes and fibrocytes from bone marrow. The combination of these events leads to an increased expression and secretion of elastin, collagen, proteoglycans and glycoproteins, causing the accumulation of the ECM and therefore the fibrotic process. This represents a prerequisite for cirrhosis and cancer.

Liver fibrosis is a pathological process that leads to an excessive accumulation of extracellular matrix (ECM) proteins and the loss of the physiological liver tissue architecture [1]. The scar tissue produced in excess and/or not appropriately recovered alters the architecture and limits the proper functioning of the liver (Figure 1). This process is caused by different and repeated insults to the liver like chronic inflammatory lesions, chronic viral hepatitis (hepatitis B or C), bacterial (brucellosis), fungal and parasitic infections (echinococcosis), inborn errors of metabolism, lipid accumulation, iron overload, intense and prolonged use of some drugs (methotrexate, isoniazid, oxyphenisanthin, methyldopa, chlorpromazine, tolbutamide, and amiodarone), and exposure to toxic agents (alcohol) (Figure 2).

There is also a congenital form of hepatic fibrosis, an autosomal recessive disorder that primarily affects the hepatobiliary and renal system [2]. Liver fibrosis can regress in the initial stages, if the causative agent is removed, on the contrary, it can lead to cirrhosis and liver failure [3,4]. Among the pathological factors, chronic hepatitis C and B infection and alcoholic and non-alcoholic steatohepatitis are the main causes of chronic progressive liver disease, leading in 20–40% of cases to the onset of liver cirrhosis. From the anatomic-pathological point of view, cirrhosis is an advanced stage of fibrosis, characterized by the formation of regenerative nodules of hepatocytes separated by fibrous septa [4]. The cirrhotic process is associated with the necrosis of hepatocytes, the collapse of the reticular support system with consequent deposition of connective tissue, the subversion of the vascular bed, and the nodular regeneration of the remaining parenchyma. This process can take years or decades to develop, therefore a correct staging and an adequate pharmacological therapy of fibrosis are extremely important, not only for the individual prognostic evaluation of the patient, but also for the prevention of disease progression towards cirrhosis and hepatobiliary cancer [5,6].

Therefore, hepatic fibrosis has become a global health problem, associated with a high morbidity and mortality due to the development of several complications, such as portal hypertension and/or consequences such as spontaneous bacterial peritonitis, hepato-renal syndrome, hepato-pulmonary syndrome, cirrhosis and hepatocellular carcinoma [7,8].

To date, liver biopsy is the gold-standard method for the diagnosis of liver fibrosis. The histological examination is useful both to identify the etiology of the liver pathology, to establish the extent of the necrotic-inflammatory lesions and to evaluate the stage of the disease towards cirrhosis. In this scenario, particular attention should be paid to profibrogenic growth factors, cytokines, mediators and cells upstream to the activation of hepatic stellate cells (HSCs) [9]. This review provides the “state of the art” on molecular mechanisms underlying liver fibrosis and the pharmacological potential of natural sulfur-containing compounds as an alternative therapeutic strategy, in order to define new directions in the fight against chronic liver diseases. Since these compounds are powerful antioxidants, due to the unique chemical properties of sulfur atoms, the relationship among reactive oxygen species (ROS)/nitric oxide (NO) production, liver fibrosis and the protective role of sulfur compounds, has been highlighted.

## 2. Molecular Mechanisms Underpinning Liver Fibrosis

It is well recognized that elevated intracellular concentrations of ROS induce redox imbalance in the liver and are involved in the apoptotic and necrotic process of the hepatocytes. ROS can stimulate the production of pro-fibrogenic mediators by Kupffer cells and the recruitment of circulating inflammatory cells, leading to direct activation of the HSCs [10], involved in the onset of the fibrotic process and its reversion [11,12]. While oxidative stress is a key player in triggering liver damage, finely regulated levels of glutathione (GSH), the most abundant antioxidant thiol in the cell, contribute to maintaining a healthy liver, preserving its functionality [13]. GSH is synthesized inside the cell and partially released in the extracellular space along a gradient concentration. In the extracellular space, GSH is hydrolyzed by γ-glutamyl transpeptidase (GGT), a dimeric enzyme located on the membrane surface [14]. GGT is highly expressed in the liver, where it is responsible for GSH metabolism and detoxification mechanisms [14,15], together with glutathione *S*-transferases (GSTs), a family of phase II metabolic isozymes, known for their ability to catalyze the conjugation of the reduced form of GSH—via the sulfhydryl group—to electrophilic centers of xenobiotic substrates, making them more water-soluble and more sensitive to detoxification [16].

**Figure 3:**
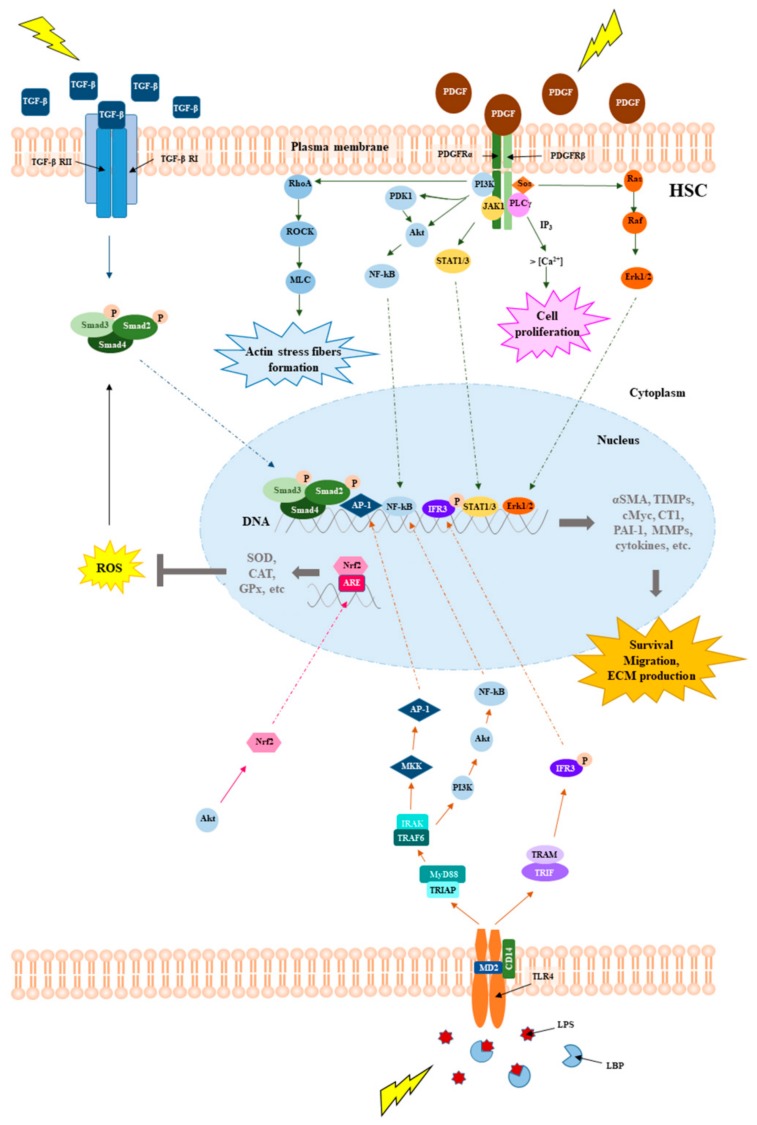
Schematic representation of the main molecular pathways underlying chronic liver disease. Comparison of the main molecular pathways involved in the pathogenesis of liver disease: TGF-β, PDGF and TLR4 signaling.

Oxidative stress in the liver plays a key role in the activation of the pleiotropic cytokine transforming growth factor beta 1 (TGF-β1). TGF-β1 is secreted as a latent complex, and only following cleavage (by proteases), denaturation (by pH modifications), or structure modification (by ROS) of the associated protein latency associated peptide, it can interact with the receptor and activate the TGF-β1 dependent signaling cascades. The TGF-β1 pathway strongly contributes to HSCs activation by modulating the expression and secretion of numerous proteases and their regulators (Figure 3) [17]. TGF-β1 can also self-induce its production thus amplifying its functions [18]. In detail, the TGF-β pathway is activated upon the interaction of type II TGF-β receptor (TGF-β RII) with the ligand. This interaction triggers the phosphorylation of type I TGF-β receptor (TGF-β RI), thus stimulating a signal cascade of phosphorylation events on small mother against decapentaplegic proteins (SMAD) leading to the formation of a complex, which migrates into the nucleus and acts as a transcription factor of several fibrogenic genes. ROS play key roles also in mediating concomitant events, such as inflammation and lipid metabolism dysregulation. These events can be activated by stressful insults including radiations and lead to the rising of the fibrogenic process, mainly through the interplay between the TGF-β pathway and wingless/integrated (Wnt) signaling, which positively regulate each other, while PPAR γ expression decreases and fails to reduce TGF-β for the opposite interplay of the canonical Wnt/β-catenin pathway [19,20]. HSCs proliferation, differentiation and migration can also be regulated by the platelet-derived growth factor (PDGF) signaling [21]. Following the interaction with the eterodimeric receptor, PDGF signaling activates a variety of downstream cascades, including phosphatidylinositol 3-kinase (PI3K)/protein Kinase B (Akt), ras homolog gene family, member A (RhoA)/Rho-associated protein kinase (ROCK), janus kinase 1 (JAK)/signal transducers of activated transcription (STAT), Ras/Raf leading to the activation of several important transcription factors as the nuclear factor kappa-light-chain-enhancer of activated B cells (NFkB), STAT1/3 and extracellular signal-regulated kinases 1 and 2 (Erk1/2), which enhance the expression of genes involved in survival, migration and ECM production.

The toll like receptor 4 (TLR4) pathway can additionally mediate the inflammatory response, fibrogenesis and survival processes [22] (Figure 3). The impairment of the intestinal barrier, presumably caused by the alteration of the gut microbioma, can allow the microbes to reach the liver through the hepatic portal system, and lipopolysaccharide (LPS) can activate the TLR4 signaling. The activated downstream cascades, myeloid differentiation primary response protein (MyD88)/mitogen-activated protein kinase kinase (MKK)/PI3K and TIR-domain-containing adapter-inducing interferon-β (TRIF)/interferon regulatory factor 3 (IFR3) contribute to the onset of chronic liver disease.

Once HSCs are activated, they show an increase in proliferation, migration, and contact-ability as well as an increase in resistance to apoptosis. At the molecular level, they show a greater expression of smooth muscle actin (α-SMA) and procollagen-I [23], which promote the ability of activated HSCs to deposit collagen and other proteins of matrix in the extracellular space. Furthermore, the activation of HSCs implies an altered regulation of matrix remodeling, involving enzymes such as tissue transglutaminase (tTG), matrix metalloproteinases (MMPs), and their inhibitors (TIMPs) [24]. Among the other factors affecting liver fibrosis is endothelin 1 (ET-1), a peptide widely distributed in the liver, which induces HSC proliferation [25] and the decrease of insulin-like growth factor (IGF), involved in the differentiation, proliferation, and apoptosis of hepatocytes [26]. Consequently, all the cell types, the signaling pathways, and the molecules that play a critical role for the appearance, progression, and reversal of fibrosis can be considered targets for a possible therapy.

## 3. Natural Sulfur-Containing Compounds for the Treatment of Liver Fibrosis

Despite the increasing understanding of the molecular landscape underlying the pathogenesis of chronic liver diseases, including the inflammation and fibrosis processes, to date there are no effective treatments for hepatic diseases. The removal of the causal agent, such as viruses and parasites but also drugs/toxins, represents the first line of defense against liver related pathologies, but this is often not sufficient to cure advanced stages of fibrosis. The main therapeutic strategies include ROS scavenging, inhibition of hepatic damage, anti-inflammatory action, deactivation and elimination of the cells responsible for the ECM production and degradation, inhibition of cytokine signaling, restoration of the intestinal microbioma, and restoration of the blood flow [27]. In addition to the standardized clinical treatment guidelines from professional associations such as the American Association for the Study of Liver Disease or the European Association for the Study of the Liver, complementary and/or alternative therapies, including the use of natural compounds can offer a good strategy in the cure against chronic liver diseases [27]. In this context, it is notable to identify liver specific bioactive compounds, with negligible immunogenicity and side effects, produced in great amounts at low costs [28,29]. There is a large number of bioactive compounds with documented anti-inflammatory and anti-fibrotic properties, including molecules isolated from the marine environment, although only few marine compounds are currently accepted as drugs in the market [30,31]. Several studies have shown the efficacy of different natural products and phytochemicals present in food and used as food extracts (such as sulforaphane, S-allycysteine, curcumin, proanthocyanidins, garlic extract, ovothiol, coffee, grape skin, or seeds) for preventing or reducing the progression of liver fibrosis with different mechanisms of action and in different animal models. In particular, the use of the numerous antioxidants containing sulfur, able to induce an appreciable/consistent reversion of the fibrotic phenotype in murine models of liver fibrosis, should be highlighted [32,33,34,35,36,37,38].

Sulfur containing low molecular weight compounds are widespread in nature from bacteria to plants, fungi, and animals, playing essential roles in the biology of the cells. Indeed, sulfur atoms can be involved in a great variety of reactions due to their unique chemical properties, as the high number of oxidation states which can form different chemotypes with different functions, including redox activity, metal-binding, and catalysis. In particular, sulfur redox activities are ascribed to its great reactivity against ROS and NO, through the formation of radicals, preventing from oxidative damage, and nitrosothiols, key mediators in NO signaling, having a physiological and therapeutic impact in many tissues, including liver [39,40]. Then, the properties of sulfydryl groups confer major biological activities to the sulfur-containing natural products [41,42]. The relevance of sulfydrilic groups in therapeutic compounds is also highlighted by the finding that a gas as hydrogen sulfide (H_2_S) regulates fundamental processes in the liver, such as mitochondrial functionality, sensitivity to insulin, and macronutrients metabolism, and is involved in the pathogenesis and treatment of several liver diseases, including hepatic fibrosis, cirrhosis, and cancer [43].

### 3.1. Glutathione

**Figure 4:**
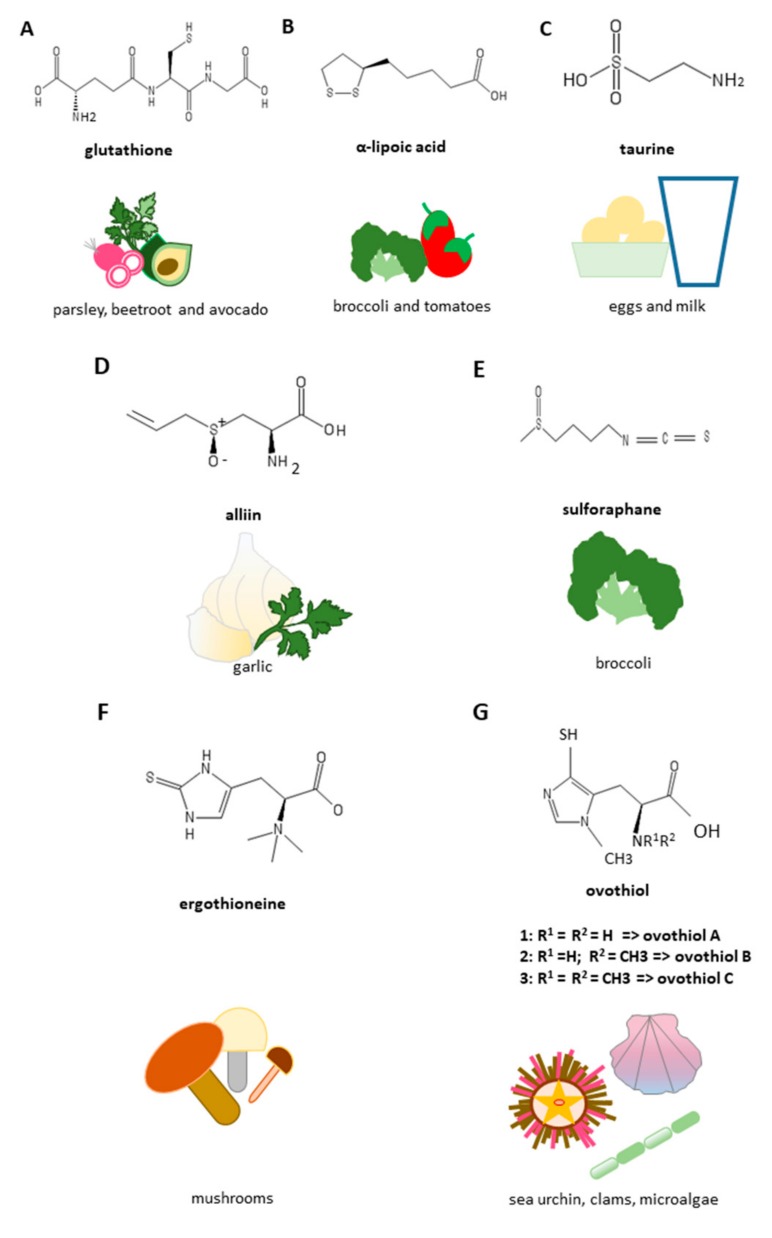
Structures and richest food sources of anti-fibrotic sulfur-containing compounds. (**A**) Glutathione; (**B**) α-lipoic acid; (**C**) taurine; (**D**) alliin; (**E**) sulforaphane; (**F**) ergothioneine; (**G**) ovothiol.

The most important and widespread low molecular weight sulfur-containing compound in the cells of plants, animals, fungi, bacteria, and archaea is GSH. It is a tripeptide composed of three amino acids: L-cysteine, L-glutamic acid, and glycine, characterized by an unusual gamma peptide linkage between the carboxyl group of the glutamate side chain and the α-amino-group of cysteine (Figure 4A). This unusual gamma amide linkage protects GSH from the hydrolysis by peptidases in the blood [15]. In animal cells, GSH ranges from 0.5 to 10 mM and it is present both in the cytosol and the organelles, both in the reduced (GSH) and oxidized form (GSSG). In the reduced form, the thiol group of cysteinyl residue is able to donate a reducing equivalent to an oxidized acceptor, thus acting as an antioxidant. In healthy cells and tissues, the ratio of GSH versus the disulfide form GSSG is in favor of the reduced form to maintain the reducing intracellular environment and the correct functioning of proteins and enzymes [44]. Therefore, an increased GSSG/GSH ratio is indicative of oxidative stress. GSSG can be converted to the reduced state by glutathione-reductase using NADPH as acceptor of electrons.

Besides neutralizing radicals and peroxides, GSH participates in thiol protection and redox regulation of cellular proteins under oxidative stress by protein S-glutathionylation, a redox-regulated post-translational thiol modification, as well as it represents a natural storage of intracellular NO through the formation of S-nitrosoglutathione [44,45,46]. Although all animal cells produce GSH, its synthesis in the liver has been shown to be essential. Indeed, knockout mice of the enzyme responsible for GSH synthesis die within a month of life due to the absence of hepatic GSH production. Reduced levels of GSH in the cell lead to the appearance of liver diseases. In particular, GSH treatment has shown very promising recovering activities from liver damage induced by oxidative stress in alcohol and non-alcoholic liver diseases [13]. It is not surprising that GSH has been tested as supplement for its detoxification and antioxidant/ROS scavenging activities, being the oxidative stress one of the main pathogenic factors in liver diseases. Parsley, beetroot, spinach, avocados, and asparagus are some of the richest dietary sources of GSH. However, dietary GSH is poorly absorbed by the human body due to the absence of a specific carrier on cell membranes [47]. Although oral administration of GSH improves hepatic metabolism in patients with non-alcoholic liver disease (NAFLD), systemic bioavailability of GSH is low, while the direct administration as intravenous injection in patients suffering from liver disease, results in a significant improvement of liver function even several months after stopping treatment. Since direct supplementation of GSH is not always successful, supply of its sulfur amino acid component, i.e. cysteine, or its derivatives may be more effective at increasing GSH levels. For example, carbon tetrachloride (CCl_4_)-induced liver fibrosis is attenuated in rats following administration of selenium-glutathione-enriched probiotics by up-regulating the hepatic silent information regulator 1 (SIRT_1_) and the consequent activation of the signaling cascade, leading to reduction of oxidative stress and endoplasmic reticulum (ER) stress, as well as to the inhibition of mitogen-activated protein kinase (MAPK) signaling [48]. GSH structurally related compounds, as N-acetyl-L-cysteine (NAC), S-Nitroso-N-acetylcysteine (SNAC), S-adenosyl-L-methionine (SAM), and S-allylcysteine (SAC) have been used in clinics to treat liver fibrosis, being precursors for glutathione synthesis and also possessing hepatoprotective roles (reviewed in [30]).

### 3.2. Lipoic Acid

Lipoic acid (or α-lipoic acid, ALA) is a natural organosulfur compound, introduced through the diet by consuming broccoli, tomatoes, spinach, salads, cabbage, peas, brewer’s yeast, brown rice, and meat (Figure 4B) [49]. Unlike other antioxidants, it can be both fat-soluble and water-soluble, therefore it acts and performs its function on a considerable number of free radicals, both inside and outside the cell. It possesses two sulfur atoms that form a redox-sensitive disulfide bond, and several studies have reported ALA antifibrotic properties. Indeed, the reduced form of dihydrolipoic acid has been shown to modulate the ROS-triggered signaling in previously activated HSCs during thioacetamide-induced hepatic fibrosis [50]. In a rat model of bile duct ligation (BDL)-induced liver fibrosis ALA downregulated the hepatic inhibitor-1 of the plasminogen activator inhibitor-1 (PAI-1) expression by inhibiting the TGF-β signaling mediators, like Smad3, activator protein-1 (AP1), and specificity protein 1 (Sp1) [51]. Yet, in a CCl_4_-induced liver fibrosis model ALA prevented collagen deposition and oxidative stress, as well as it modulated the expression of the pro-inflammatory cytokine interleukin-6 (IL-6), the inducible nitric oxyde synthase (iNOS), the NF-kB, and the MMP-13 [52]. ALA also provided protection from liver ischemia-reperfusion injury [53] or from tissue damage induced with different treatments, including lipopolysaccharide/D-galactosamine/methotrexate injection [54,55,56,57,58,59], bisphenol-A oral administration [60], a high fat or fructose rich diet [61,62,63] or methionine–choline deficiency [64]. Furthermore, the treatment with ALA of rats with hepatic fibrosis resulted in a reduction in the activity of aspartate transaminase (AST) and alanine transaminase (ALT), improveed hepatic injury by decreasing the deposition of collagen fibers, and restoreed the Akt/mammalian target of rapamycin (mTOR) pathway. All this is possible thanks to its ability to inhibit the TGF-β/Smad3 pathway with consequent suppression of autophagy [65]. In addition, clinical trials consisting in ALA supplementation in patients with NAFLD have reported a modulation of some of the oxidative stress related parameters, including serum malondialdehyde levels and total antioxidant status, as well as a modification of the adipokine profile, known to play a key role in the progression of NAFLD [66,67].

### 3.3. Taurine

Taurine is an amino sulfonic acid, also referred as an essential amino acid for many species, while for humans is conditional. We basically assume taurine by diet. It is present in eggs, fish, meat, and milk, but not in foods of plant origin, and seafood is considered the richest source (Figure 4C). Taurine-based supplements have also been developed due to several therapeutic applications [68]. In adults, taurine can be synthesized in the liver starting from cysteine and methionine in the presence of sufficient amounts of vitamin B6. Taurine is involved in many cellular biological activities such as bile acid conjugation, cell membrane stabilization, and calcium signaling. *In vitro* and *in vivo* studies reported that taurine treatment of HSCs isolated from healthy rats significantly inhibited proliferation, reduced ROS dependent damage, like lipid peroxidation, and provided protection from fibrogenesis, while rats with CCl_4_-induced liver damage supplemented with taurine were protected from liver histological damage and fibrosis, showing a decrease of oxidative stress markers and fibrogenic factors [69,70]. In addition, taurine protective action seems to be especially displayed in the pericentral region of the liver, where taurine transporter is mostly expressed and this amino acid may be useful against xenobiotics-induced hepatic damages presumably interfering with the NADPH dependent cytochrome P450 2E1 catabolic activity [71]. *In vivo* studies have reported that taurine administration provides protection against alcohol-induced liver damage in rats by lowering the levels of circulating transaminases and inflammatory cytokines and increasing the hepatic total protein and antioxidant defense, as well as by modulating HSC activation and collagen deposition [72,73]. More recently, the molecular mechanisms of the taurine hepatoprotective activity against alcohol induced damage has been clarified and, in particular, the inhibition of TLR4/MyD88 and NF-κB signaling have been identified as the target pathways [74]. Taurine administration also accelerates alcohol and lipid metabolism in alcoholic fatty liver disease [75]. In addition, taurine reduces ROS damage and increases antioxidant defense in liver previously injured by iron-overloading, resulting in anti-fibrotic and anti-apoptotic properties [76,77]. Moreover, taurine has the ability to attenuate portal hypertension in rats with induced cirrhosis and in cirrhotic patients by acting on vasodilatation through the regulation of NO synthesis [78,79]. Yet, the therapeutic potential of taurine is extended to the diet induced NAFLD showing anti-apoptotic and antioxidant activities in in vitro and ex vivo models and, in addition, a modulation of energetic metabolism in in vivo experiments [80].

### 3.4. Garlic Derived Sulfur Compounds

Garlic (*Allium sativum*) is a member of the family Liliaceae, having a huge number of bioactive properties mainly due to its content in organosulfur compounds, which are able to regulate the expression of a wide variety of genes, including the inducible NOS, with a strong impact for human health [81]. The main sulfur compound present in fresh garlic is alliin (Figure 4D) which is metabolized to allicin upon the action of the enzyme allinase when garlic is crushed or cut. Allicin is then metabolized in other compounds, including diallyl sulphide (DAS), di- (DADS) and trisulfide. Intraperitoneal injections of garlic extract in rats with CCl_4_-induced liver damage elicit an anti-fibrotic effect by modulating HSCs activation, increasing degradation of ECM and leading to liver tissue regeneration and the re-establishment of its function mainly through inhibition of TGF-β1 signaling pathway [33]. SAC, a key component of aged garlic extract, has shown anti-fibrotic properties in liver-damaged rats previously injected with porcine serum, mainly through inhibition of HSCs activation [36]. SAC has been shown to be an endogenous donor of H_2_S, which plays key roles in the gastrointestinal tract and in the liver. It has been reported that SAC decreases the expression of transaminase levels in rats in which hepatic damage was induced by treatment with CCL_4_, at the same time leading to a decrease in the levels of gene expression of the main actors of liver damage, such as TGF-β1, tTG, α-SMA, fibronectin, and collagen I [33]. An interesting property of garlic extract is the ability to block the enzymatic activity of tTG; this prevents the covalent stabilization of the fibrils making the matrix more resistant to degradation by specific collagenases [32]. Furthermore, SAC reduced the phosphorylation of SMAD3, signal transducers and transcriptional activators and further inhibited their ability to bind to transcription promoters. Taken together, SAC administration attenuated CCl4-induced liver fibrosis in rats with antioxidant, anti-inflammatory, and antifibrotic effects targeting the STAT3/SMAD3 pathway to inhibit gene transcription [82]. DADS showed protective properties from Trichloromethane-induced liver damage in rats by inhibiting NFkB activation and apoptotic processes and increasing the antioxidant hepatic defenses [83]. Moreover, the treatment with S-allyl-glutathione (SAG) of mice with hepatic fibrosis leads to a dose-dependent decrease in heat shock protein-47 (Hsp47), a specific collagen chaperone and other fibrosis markers. SAG acts also directly on a mannose receptor present on Kupffer cells (hepatic macrophages), thereby indirectly inhibiting the activation of HSCs [84].

The sulfur atoms in these molecules seem to be important for the liver functioning. Indeed, the three major sulfur containing garlic components (diallyl sulfide, disulfide, and trisulfide) have been shown to have a stronger modulatory activity on the hepatic detoxification system along with the increase of the number of sulfur atoms [85]. Many other studies have reported the hepatoprotective properties of garlic and garlic-derived compounds mainly thanks to their ROS scavenging and anti-inflammatory activities [86].

### 3.5. Sulforaphane

Sulforaphane (SFN) is a dietary isothiocyanate obtained by the enzymatic processing of glucopharanin, a 4-methylsulfinylbutyl glucosinolate, present in cruciferous vegetables, as broccoli and cabbage (Figure 4E). SFN has been shown to protect from BDL-induced liver fibrosis in mice by modulating the nuclear factor erythroid 2-related factor 2 (Nrf2)-mediated inhibition of the TGFβ/Smad signaling pathway, with consequent inhibition of HSCs activation and fibrosis [34]. SFN oral administration had hepatoprotective properties in mice with CCl_4_-induced liver damage leading to a decrease of ALT serum levels, inhibition of necrotic processes and ROS-induced lipid peroxidation, as well as to an induction of phase 2 detoxification enzymes, including GST [87]. SFN also provides protection from hepatotoxicity induced with a variety of insults, such as cisplatin and LPS, preserving mitochondrial functioning [88] and targeting specific pathways, including MyD88 and anti-TLR-associated activator of interferon (TRIF)-dependent signaling pathways of the TLR system [89]. Feng et al. [90] have investigated the possibility that SFN could inhibit the HSC activation by interfering with microRNAs, which play a key regulative role in the development of liver fibrosis. They demonstrated that SFN is able to downregulate miRNA-423-5p in LX-2 primary human HSC line interfering with their activation. Interestingly, a preclinical phase study reported that CCl_4_-induced liver fibrosis in rats was prevented by the combination of capsaicin (CPS) and SFN via oral gavage by up-regulating PPARγ and Nrf2 [91]. The doses they used were safe for consumption and could be easily assumed with foods in humans, suggesting the use of functional foods containing these bioactive compounds (chili peppers for CPS and, as mentioned before, cruciferous vegetables for SFN) for prevention of hepatic diseases, although further studies are needed to find the optimal concentrations for use in humans.

### 3.6. Sulfur-Containing Histidines

Sulfur-containing histidines are amino acid derivatives containing a thiol group on the imidazole ring. They can be distinguished based on the position of such substitution in 2-thiohistidines and 5-thiohistidines. Ergothioneine (ERG), first identified from the ergot fungus *Claviceps purpurea*, is a trimethyl-L-histidine, characterized by the thiol group on the carbon 2 of the imidazole ring of histidine and three methyl groups on the side chain. The biosynthesis of ERG has been only observed in Actinobacteria (for example, Mycobacterium spp.) and some fungi. In humans it is obtained from dietary sources, mainly mushrooms, and can be accumulated in human tissues such as liver and blood, spleen, kidneys, lungs, heart, intestine, at relatively high concentrations (Figure 4F) [92,93], thanks to its high affinity to the organic cation transporter 1 (OCTN1) [94]. Indeed, the silencing of OCTN1 encoding gene has been shown to prevent ERG absorption in murine tissues [95]. The physiological functions of ERG have not been fully clarified yet. However, several *in vitro* studies have shown the ability of ERG to eliminate hydroxyl radicals, hypochlorous acid, peroxynitrite [96], singlet oxygen [97], modulates inflammatory responses [98,99,100] and protects against UV and gamma rays [101].

ERG accumulation in tissues suffering a high oxidative stress situation has been proposed to have a protective role. Indeed, induced OCTN1 upregulation and consequent ERG increasing uptake and accumulation in liver of a guinea pig model of NAFLD have been proved to exert hepatoprotective roles both by an antioxidant action, through hsp70 modulation, and by chelating Fe^2+^ with consequent inhibition of Fenton chemistry [102]. The hsp70 upregulation by ERG has resulted in being protective also for liver injured by ischemia reperfusion leading to a reduction of lipid peroxidation [103]. Moreover, functional studies have demonstrated that OCTN1 expression has a protective role in in vitro and in vivo liver fibrosis models through ERG uptake regulation and consequent gene downregulation of NADPH oxidase 4, a NOX isoform highly expressed in hepatocytes and HSCs, with reduction of oxidative stress and fibrosis [104].

Ovothiols are methyl-5-thiohistidines, having the thiol group in position 5 and the methyl group in position 2 of the imidazole ring of histidine. They can be distinguished in three forms based on the presence/absence of additional methyl groups on the nitrogen group of the side chain: ovothiol A with no additional methyl groups, ovothiol B having one methyl, ovothiol C having two methyl groups [105]. Ovothiols can be synthesized starting from cysteine and histidine in the presence of iron and oxygen thanks to the catalysis of the sulfoxide synthase OvoA and the beta-lyase OvoB [106,107,108]. They are powerful antioxidants, widespread in the marine environment, and most abundant in sea urchin eggs, the richest food source available [109] (Figure 4G). Other marine sources of ovothiols are clams, holoturians, and microalgae [108]. The unique position of the sulfur atom in these molecules confers them a stronger ROS scavenging activity compared to other thiols, like GSH and ERG, protecting also from hydroperoxides and peroxynitrite-induced damage [105]. Ovothiol A has been shown to induce autophagy in a human liver cancer cell line, HepG2 [110]. It also inhibits collagen deposition in a CCl_4_-induced liver fibrosis murine model, by modulating fibrogenic markers, such as TGF-β, α-SMA, and TIMP-1, as well as by regulating the activity of GGT, a key enzyme involved in the evolution of liver fibrosis [38]. In particular, ovothiol directly inhibits membrane bound GGT activity in human cancer cells, including hepatocarcinoma cells [111]. Consequently, ovothiol exerts its hepatoprotective action by regulating GGT activity and GSH levels.

## 4. Conclusions and Perspectives

To date there are no drugs for the treatment of liver fibrosis whose causes can be different, mainly chronic alcohol or drug abuse, viral hepatitis B or C, nonalcoholic fatty liver disease (NAFLD), nonalcoholic steatohepatitis (NASH), a subtype of NAFLD. Therefore, the best way to reduce liver fibrosis consists in eliminating alcohols and drugs, treatment with antiviral drugs for hepatitis B and C, and an appropriate nutrition and lifestyle in the case of NAFLD and/or NASH. Integrating the diet with natural compounds, that have demonstrated antifibrotic properties, and their pharmacological administration, together with antiviral treatment of hepatitis or other insult-specific therapies, can be considered valid therapeutic strategies to regress fibrosis that otherwise could have much more serious consequences. For example, there are several clinical trials for patients with NAFLD that involve the oral administration of sulfur-containing compounds such as GSH, ALA, and taurine [47,66,67,79].

**Figure 5:**
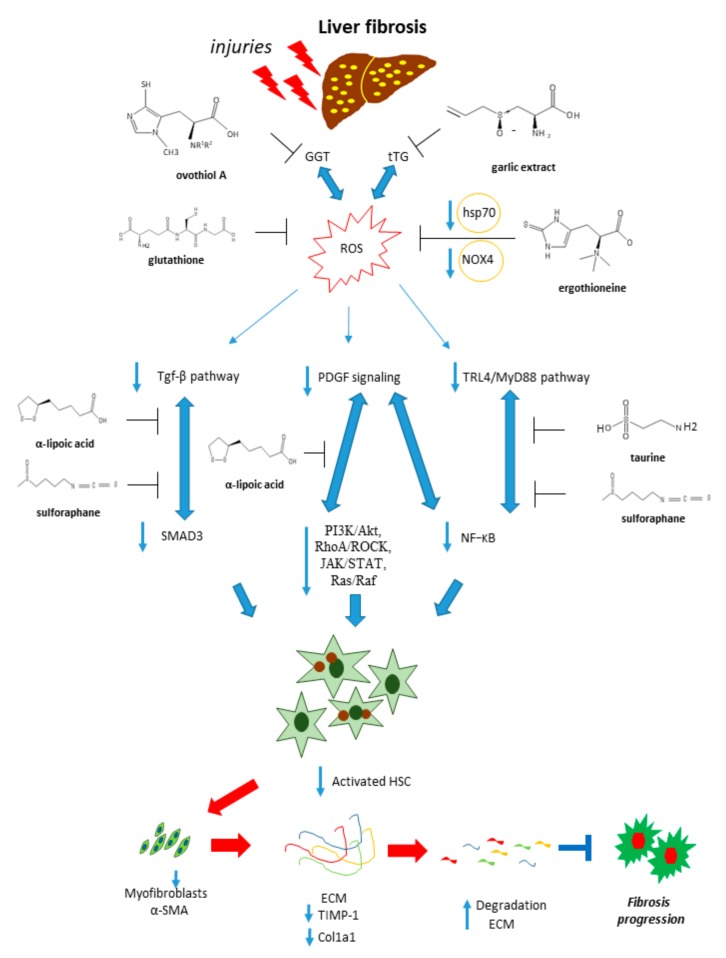
Antifibrotic properties of sulfur-containing compounds. Sulfur-containing compounds are able to interfere with molecular pathways associated with liver fibrosis, such as TGF-β, PDGF, and TLR4/MyD88 signaling, leading to the inhibition of HSCs activation and the fibrogenesis process.

These compounds are pleiotropic molecules acting as ROS and NO scavengers, thiol/disulfide exchange modulators. Thanks to these properties they can regulate several redox-sensitive pathways like TGF-β, PDGF, and TLR pathways, involved in the progression of fibrosis (Figure 5).

Some of these natural sulfur compounds can be efficiently acquired by diet through specific membrane transporters as known for ERG and taurine. Others, such as ovothiols of marine origin, have been only recently receiving attention as more potent antioxidants and anti-inflammatory compounds. Several patents have been developed to treat and prevent liver fibrosis through the supplementation of sulfur-containing molecules (Table 1). The most effective compounds seem to be ALA, SAC, and SFN. Ovothiol exerts its anti-fibrotic activity in mice affected by liver fibrosis at doses comparable to ALA, SAC and SFN (Table 1). Future studies need to explore the potential of these small sulfur-containing compounds as dietary supplements and/or new marine drugs for the treatment of liver diseases in humans. Moreover, the discovery of new natural sulfur-containing molecules in the future would help to face the challenge for advanced clinical trials. We expect the discovery of such novel compounds from the ocean, which has recently attracted the interest of the scientific community as a still poorly explored source of food and medicines for mankind.

## Figures and Tables

**Table 1:** Therapeutic efficacy of sulfur-containing compounds in liver fibrosis models. The name of compounds, the experimental models, the doses, and the related patents are reported.

Compound	Model System	Concentration	Patent
Glutathione (GSH)	Wistar rats with CCl_4_-induced liver fibrosis	34.1 mg/g [43]	*n* = 5 US201816232747 CN201811444979 CN201710216398 WO2011US23434 KR19920005534
Lipoic acid (ALA)	C57BL/6 mice with BDL-induced liver fibrosis	100 mg/kg [46]	*n* = 1 WO2017KR15370
	Wistar rats with CCl4-induced liver fibrosis	30 mg/kg [47]	
Garlic derived sulfur compounds (SAC, DADS, SAG)	Rat with CCl_4_-induced liver fibrosis	200 mg/kg (SAC) [30,31]	*n* = 1 JP20050270344
	Wistar rats with porcine serum-induced liver fibrosis	0.15% of diet (SAC) [34]	
	Rat with CCl_4_-induced liver fibrosis	50 mg/kg (SAC) [77]	
	Rat with trichloromethane (TCM) induced liver fibrosis	50 mg/kg (DADS) [78]	
	Rat with CCl_4_-induced liver fibrosis	200 mg/kg (SAG) [79]	
Ergothioneine (erg)	Guinea pig model of NAFLD	4%–10% of diet [97]	*n* = 1 USOO6555141B1
Ovothiol (ovo)	Male balb-c CCl_4_-induced liver fibrosis	50 mg/kg [36]	*n* = 2 WO2019/08247A1 RBI15865-IT

## Abbreviations

Akt	Protein Kinase
ALA	α-lipoic acid
ALT	Alanine transaminase
AP-1	Activator protein-1
AST	Aspartate transaminase
BDL	Bile duct ligation
cMyc	Cellular myelocytomatosis
CPS	Capsaicin
DADS	Diallyl disulphide
DAS	Diallyl sulphide
ECM	Extracellular matrix
ERG	Ergothioneine
Erk1/2	Extracellular signal-regulated kinases 1 and 2
ET-1	Endothelin-1
GGT	γ-glutamyl transpeptidase
GPx	Glutathione peroxidase
GSH	Glutathione
GST	Glutathione S-transferases
H_2_S	Hydrogen sulfide
HSC	Hepatic stellate cell
Hsp	Heat shock protein
IFR3	Interferon regulatory factor 3
IGF-1	Insulin-like growth factor 1
IL-6	Interleukin-6
iNOS	Inducible nitric oxide synthase
LBP	LPS-binding protein
LPS	Lipopolysaccharide
MAPK	Mitogen-activated protein kinase
MKK	Mitogen-activated protein kinase kinase
MMPs	Matrix metalloproteinases
mTOR	Mammalian target of rapamycin
MyD88	Myeloid differentiation primary response protein
NAC	N-acetyl-L-cysteine
NAFLD	Non-alcoholic liver disease
NF-κB	Nuclear factor kappa-light-chain-enhancer of activated B cells
NOX	NADPH oxidase
Nrf-2	Nuclear factor erythroid 2-related factor 2
OCTN1	Organic cation transporter 1
PAI-1	Plasminogen activator inhibitor-1
PDGF	Platelet-derived growth factor
PDGFRα/β	Platelet-derived growth factor receptor isoform α/β
PDK1	Phosphoinositide-dependent protein kinase 1
PI3K	Phosphatidylinositol 3-kinase
PLCγ	Phospholipase C γ
PPARγ	Peroxisome proliferator-activated receptor γ
Raf	Rapidly accelerated fibrosarcoma
RhoA	Ras homolog gene family, member A
ROCK	Rho-associated protein kinase
ROS	reactive oxygen species
SAC	S-allylcysteine
SAG	S-allyl-glutathione
SAM	S-adenosyl-L-methionine
SECs	Sinusoidal endothelial cells
SFN	Sulforaphane
SIRT1	Silent information regulator 1
SMAD2/3/4	Small mother against decapentaplegic protein 2/3/4
SNAC	S-Nitroso-N-acetylcysteine
SOD	Superoxide dismutase
Sp1	Specificity protein 1
STAT1/3	Signal Transducers of Activated Transcription 1/3
TGF-β	Transforming growth factor beta
TGF-β RI/II	Transforming growth factor beta receptor type I/II
TIMPs	Tissue inhibitors of metalloproteinases
TIRAP	TIR-domain containing adapter protein
TLR4	Toll-like receptor 4
TRAF6	Tumor necrosis factor receptor-associated factor 6
TRAM	Translocating chain-associating membrane protein
TRIF	TIR-domain-containing adapter-inducing interferon-β
tTG	Tissue transglutaminase
Wnt	Wingless/integrated
αSMA	α-smooth muscle actin
